# Efficacy and Safety of Banxia Formulae for Insomnia: A Systematic Review and Meta-Analysis of High-Quality Randomized Controlled Trials

**DOI:** 10.1155/2021/8833168

**Published:** 2021-05-26

**Authors:** Yan-Hua Lin, Cong Chen, Xiu Zhao, Yi-Fei Mao, Guang-Xin Xiang, Meng-Qi Yang, Yong-Mei Song

**Affiliations:** ^1^Institute for Literature and Culture of Chinese Medicine, Shandong University of Traditional Chinese Medicine, Jinan 250355, China; ^2^Department of Scientific Research Management, Shandong University of Traditional Chinese Medicine, Jinan 250355, China; ^3^College for Traditional Chinese Medicine, Shandong University of Traditional Chinese Medicine, Jinan 250355, China; ^4^Key Laboratory of Classical Theory of Traditional Chinese Medicine, Ministry of Education, Shandong University of Traditional Chinese Medicine, Jinan 250355, China

## Abstract

**Objective:**

To systematically evaluate the efficacy and safety of Banxia (Pinellia Tuber) formulae in the treatment of insomnia compared with those of conventional western medicines.

**Methods:**

Randomized controlled trials (RCTs) evaluating the efficacy and safety of Banxia formulae in the treatment of insomnia were searched from the following databases: PubMed, Cochrane Library, EMBASE, the China National Knowledge Infrastructure (CNKI), Chinese Scientific Journals Database (VIP), and Wanfang database. The literature collected was from the time when the databases were established to April 2020. Quality assessment and meta-analysis were conducted by using Cochrane bias risk assessment tool and RevMan 5.2, respectively. Publication bias was assessed by Egger's test.

**Results:**

Fourteen RCTs with 910 participants were identified. A total of 46 traditional Chinese medicines involving 2 different dosage forms were used in the included studies. Meta-analysis indicated that Banxia formulae had more significant effects on improving the total effective rate (*RR* = 1.23, 95% *CI* 1.16 to 1.31), Pittsburgh Sleep Quality Index (PSQI, *MD* = −1.05, 95% *CI* −1.63 to −0.47), and the TCM syndrome score (*SMD* = −0.78, 95% *CI* −1.18 to −0.39). Meanwhile, on reducing adverse events, Banxia formulae also showed an advantage (*RR* = 0.48, 95% *CI* 0.24 to 0.93).

**Conclusion:**

According to the current studies, the efficacy of Banxia formulae in the treatment of insomnia is better than that of the conventional western medicines, and its safety is relatively stable. However, due to the limitations of this study, further research and evaluation are needed.

## 1. Introduction

Insomnia is a widespread health complaint in general population which deserves appropriate recognition and attention. It is not only a risk factor for cardiovascular diseases (such as arterial hypertension, chronic heart failure, and myocardial infarction), obesity and type 2 diabetes, but also a common disease which frequently coexists with nervous system diseases (dementia, depression) [1, 2].

Cognitive behavior therapy and drug therapy are the main methods of treating insomnia. Since cognitive behavior therapy does not work for everyone in all situations, drug therapy is still the most common method for treating insomnia so far [3, 4]. In western medicine, short/intermediate acting benzodiazepines (BZ), benzodiazepine receptor agonists (BZRA), and some antidepressants are drugs of the first choice for the treatment of insomnia. Although western medicines have quick and strong effects in the treatment of insomnia, many side effects of them have been reported, including hangover, nocturnal confusion, falls, rebound insomnia, tolerance and dependency liability, withdrawal reaction, and increased risk of slight infection. [5–7]. Studies have shown that BZ and BZRA can impair one's driving ability which has significant correlation with car accidents [8–10]. Moreover, some data in current studies suggests that the mortality of people who use BZ, antidepressants, and antipsychotics is increased [11, 12]. These side effects have caused many medical and social problems.

Chinese medicine has a long history in the treatment of insomnia. Based on the conception of holism and the theory of syndrome differentiation and treatment, traditional Chinese medicine treats insomnia and regulates the patient's body as a whole. It has unique advantages in improving sleep and life quality of the patients, and has little side effect.

Banxia (Pinellia Tuber) is the dried tuber of *Pinellia ternata* (Thunb.) Makino. As a Chinese herbal medicine, Banxia has been used in the treatment of insomnia for about two thousand years. As early as in “*Huangdi*'*s Internal Classic,”* there were records of Banxia for insomnia treatment. In materia medica works in past dynasties of China, there are also records of Banxia for insomnia treatment. For example, in *“Compendium of Materia Medica,*” it said: “It (Banxia) can remove water distention and treat insomnia.” According to modern pharmacological studies, Banxia has antitussive, expectorant, antitumor, antibacterial, anti-inflammatory, antioxidant and sedative-hypnotic effects [13]. It is commonly used in the treatment of cough, vomiting, infection, inflammation, and emotional illness.

Previous meta-analysis [14] and systematic review [15] of Huanglian Wendan Decoction in the treatment of insomnia were limited by considerable risk of bias and a comprehensive meta-analysis on the efficacy of Banxia formulae in the treatment of insomnia is lacking. Therefore, the purpose of the present study was to systematically review randomized controlled trials (RCTs) of high-quality which investigate the efficacy and safety of Banxia formulae compared with those of conventional western medicines in insomnia adults.

## 2. Method

This systematic review and meta-analysis are based on the Preferred Reporting Items for Systematic Reviews and Meta-Analyses: PRISMA statement search strategy [16].

### 2.1. Search Strategy

Six databases including PubMed, Cochrane library, EMBASE, the China National Knowledge Infrastructure (CNKI), Chinese Scientific Journals Database (VIP), and Wanfang databases were searched from inception to April 2020 for the relevant RCTs of Banxia formulae for insomnia, with “insomnia” and “traditional Chinese medicine” as search terms, a subject word plus free words as search form. In order to ensure that eligible herbal formulae were included as many as possible, the specific herb name “Banxia” was not explicitly searched, and no restriction on language and publication period was set in this review. In addition, we also searched for references that have been included in relevant literature or systematic review.

### 2.2. Inclusion Criteria

1. *Type of Participants*. Adult patients diagnosed with insomnia were included. Insomnia was confirmed according to standard diagnostic criteria including the “Diagnostic and Statistical Manual of Mental Disorders, 4th edition (DSM-4)” [17] and the “Diagnostic and Statistical Manual of Mental Disorders, 5th edition (DSM-5)” [18].
2. *Type of Study.* Only RCTs that assessed the efficacy and safety of drugs for the treatment of insomnia were eligible. Trials that only mentioned “randomization” but without any description of the random allocation process were excluded.
3. *Type of Intervention*. Banxia must be included in the herbal formula used in the experimental group. There were no restrictions on the form of the drug, dosage, frequency, or treatment time. Patients in the control group were treated with conventional western medicines (BZ, BZRA, etc.).
4. *Types of Outcome Measures*. The primary outcome was the total effective rate. The secondary outcomes included Pittsburgh Sleep Quality Index (PSQI), TCM syndrome score, and adverse events.

### 2.3. Exclusion Criteria

1. Duplicate publications
2. Studies not meeting the inclusion criteria
3. Combined treatment of Banxia formulae and other therapy were used in the experimental group
4. Traditional Chinese medicine by oral administration was used in the experimental group and the control group at the same time
5. Studies with missing data
6. Studied assessed as high risk of bias by RoB2

### 2.4. Study Selection

The titles and abstracts of the retrieved articles were read by 2 independent review authors to exclude the obvious disqualified RCTs. Then, the full texts of the studies that potentially met the predefined inclusion criteria were obtained and read in order to select the eligible RCTs. When there were different opinions, the 2 review authors can reach consensus by discussing with the corresponding author of this article.

### 2.5. Quality Assessment

The methodological quality of the included RCTs was assessed by 2 independent review authors according to the Cochrane RoB2 criteria [19]. The signalling questions used to evaluate bias in each trial related to five domains: (1) bias arising from the randomization process; (2) bias due to deviations from intended interventions; (3) bias due to missing outcome data; (4) bias in measurement of the outcome; (5) bias in selection of the reported result. By responding to signalling questions, each domain was judged as low risk of bias, some concerns, and high risk of bias. Then, the overall bias of each trial was determined.

The disagreements on the methodological quality between the 2 review authors were solved by discussion and consulting a third author.

### 2.6. Data Extraction

The data extraction was performed independently by 2 review authors according to the predesigned standard data extraction forms including the following items: lead author, publication year, country of origin, characteristics of participant, course of treatment, adverse events, and outcome measures. Disagreements were resolved in line with the principle of consensus through consultation. If the study reported the outcome data at different time points, the data of the last time point was extracted. For the trials with more than 2 groups or factorial designs allowing multiple comparisons, only the information and data reported in the original articles were extracted.

### 2.7. Banxia Formulae Composition

In each included study, the Banxia formula and its composition were recorded, with a frequency analysis of common drugs combined with Banxia.

### 2.8. Data Analysis

The mean difference (*MD*) or standardized mean difference (*SMD*) was used to evaluate continuous data, and relative risk (*RR*) with 95% confidence intervals (*CI*) for dichotomous data. In order to evaluate whether the efficacy of Banxia formulae was affected by variable factors, subgroup analyses were conducted according to the course of treatment (≤21 d, >21 d), the dosage of Banxia (≤9 g, >9 g), and the processing method of Banxia (raw Banxia, processed Banxia). Based on the analysis, whether the differences among the subgroups were statistically significant was assessed. All meta-analyses in this study were performed by using the software Cochrane Collaboration Review Manage (RevMan 5.2), and *P* < 0.05 was considered statistically significant. Egger's test was carried out by Stata 15.1.

### 2.9. Heterogeneity

The statistical heterogeneity of the trials was assessed by *X*^2^ test and expressed as *I*^2^ value. When there is no heterogeneity or the heterogeneity was moderate (*P* > 0.1, *I*^*2*^ < 50%), a fixed effect model (FEM) was applied; otherwise, a random effect model (REM) was applied.

### 2.10. Publication Bias

The publication bias was identified by Egger's test, if an outcome was reported in at least 10 trials.

### 2.11. Sensitivity Analysis

The sensitivity analysis was performed by excluding one study from the meta-analysis. If the estimated value of the point exceeded the 95% *CI* of the total effect amount (or was significantly different from the combined effect amount) after the study was excluded, it indicated that there were potential risks and this study needs to be further reviewed.

## 3. Results

### 3.1. Description of Studies

**Figure 1:**
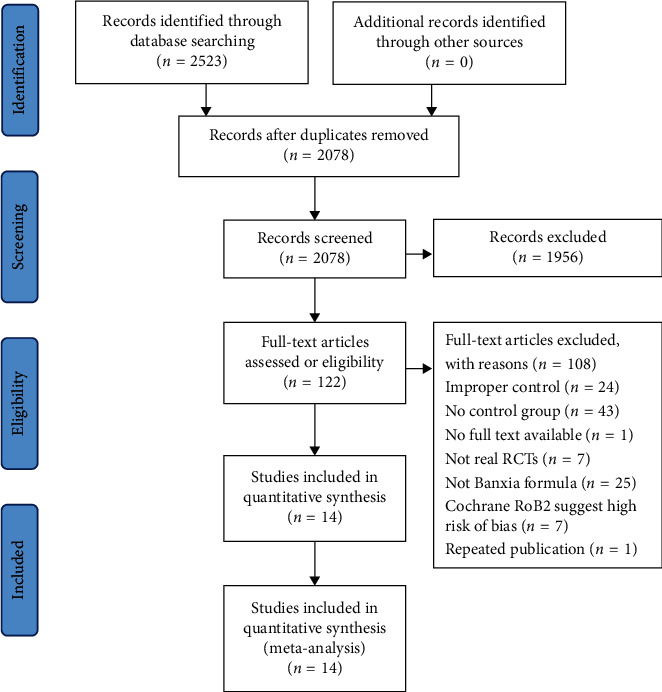
Flow diagram of literature search and selection.

A total of 2523 studies were retrieved from the 6 electronic databases. After the duplicates were removed, 1956 studies remained. In the remaining 1956 studies, 122 were eligible. By reading the full text of the 122 studies, 108 were excluded, among which 24 studies were with inappropriate interventions, 43 studies were without control group, 1 study was without full text, 7 studies were not real RCTs, 25 studies did not use Banxia formula, 7 studies had high risk of bias according to Cochrane RoB2, and 1 study was repeated publication. Finally, 14 studies including 910 participants were included and a meta-analysis was conducted on them [20–33]. A PRISMA flowed diagram shows the procedure of literature research and study selection (Figure 1).

### 3.2. Basic Characteristics of the Included Studies

Basic characteristics of the 14 included studies are summarized in Table 1. All the eligible studies were conducted and published in China. All the included studies evaluated the total effective rate, among which 11 RCTs showed that Banxia formula improved the total effective rate [20–22, 25–27, 29–33], 3 RCTs showed that Banxia formulae did not significantly improve the total effective rate compared with conventional western medicine [23, 24, 28]. In the 6 RCTs with PSQI scores, 4 showed that Banxia formula improved the total score of PSQI of patients with insomnia [22–24, 32], and the other 2 showed that Banxia formula did not significantly improve the total score of PSQI of patients with insomnia [26, 28]. In the included studies, 5 studies reported TCM syndrome score [22–24, 28, 32], and 8 studies described side effects [20, 22–26, 29, 32]. The treatment duration ranged from 7 days to 30 days (Table 1).

### 3.3. Description of Banxia Formulae

The composition of Banxia formulae in the included studies is listed in Table 2. This paper mainly introduced the basic composition of Banxia formulae in the included studies. A total of 46 herbs were used in the 14 included Banxia formulae, and 2 dosage forms were mentioned, including decoction (*n* = 13) and decoct-free granule (*n* = 1) [30] (Table 2). Among the decocting methods of the 13 decoctions, one was decocting Banxia for a long time [33] and the others were conventional decocting. The top 7 frequently used Chinese herbal medicines were Banxia (*Pinellia ternata* (Thunb.) Makino, frequency = 14), Gancao (*Glycyrrhiza uralensis* Fisch. ex DC., frequency = 10), Chenpi (*Citrus × aurantium* L., frequency = 9), Fuling (Poria cocos (Schw.) Wolf, frequency = 98), Zhuru (*Bambusa beecheyana* Munro, frequency = 8), Huanglian (*Coptis chinensis* Franch., frequency = 7), and Dangshen (*Codonopsis pilosula* (Franch.) Nannf., frequency = 6) (Table 3).

### 3.4. RoB Assessment

**Figure 2:**
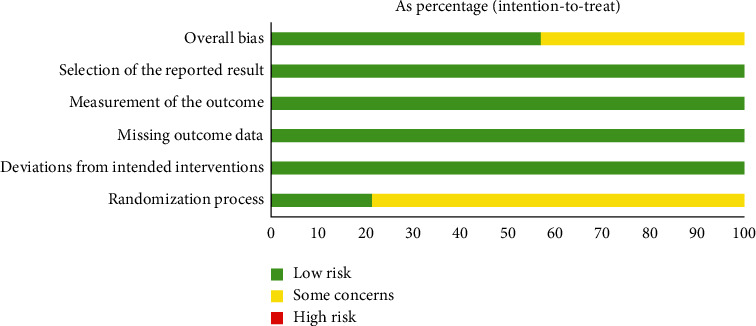
Assessment of risk of bias.

Figure 2 shows the assessment of the risk of bias. All the included studies were described as “randomized.” The random sequence was generated by random number table method (11 studies) or computer-generated random number method (3 studies). One study applied “sealed envelopes,” two studies mentioned single-blind method, and the remaining 11 studies had no clear information of blind method. There were no imbalances in the 14 studies. Therefore, there was no high risk in the randomization process. Because of vague description of blind method, 11 trials were judged as some concerns. Two studies described dropouts and provided adequate explanations, while the other 12 studies did not mention dropouts. The method of outcome measurement was suitable for the outcome they were intended to evaluate in each trial. No other significant bias was found in the included studies.

### 3.5. Efficacy Assessment

#### 3.5.1. Total Effective Rate

**Figure 3:**
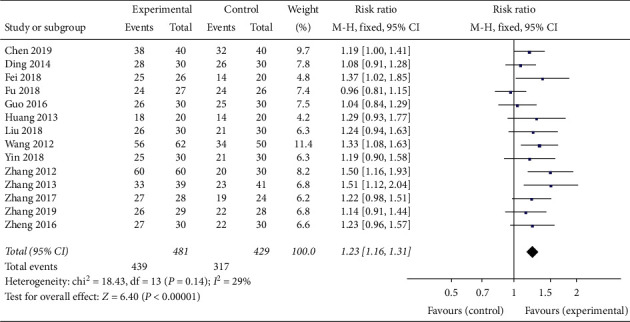
Meta-analysis of the total effective rate in experimental group and control group.

All the 14 included studies reported the total effective rate, which indicated the total effective rate of the experimental group was higher than that of the control group (*RR* = 1.23,95% *CI* 1.16 to 1.31) (Figure 3). In the heterogeneity test, *P* = 0.14, *I*^*2*^ = 29%, so the FEM was used to conduct the statistical. Pooled RR with 95% *CI* showed *Z* = 6.40, *P* < 0.00001 (Figure 3), suggesting that the difference was statistically significant. It can be concluded that the total effective rate of the experimental group with Banxia formulae was higher than that of the control group with conventional western treatment.

**Figure 4:**
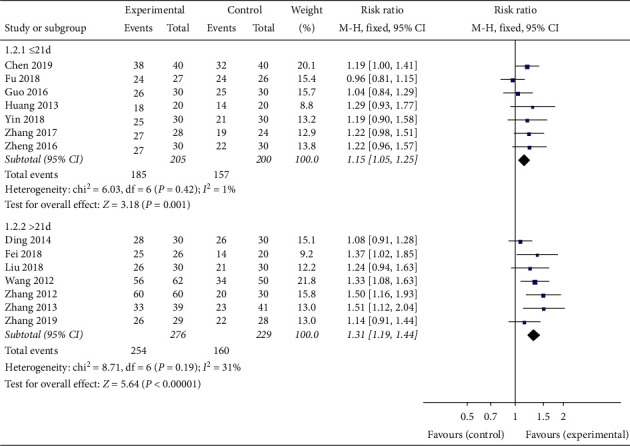
The total effective rate analysis of different duration.

**Figure 5:**
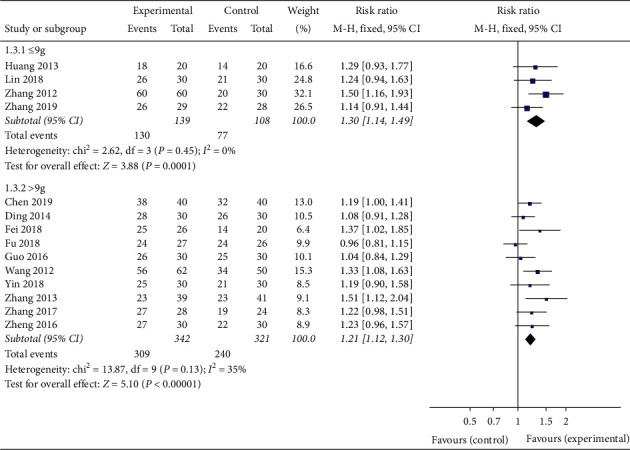
The total effective rate analysis of different dose of Banxia.

**Figure 6:**
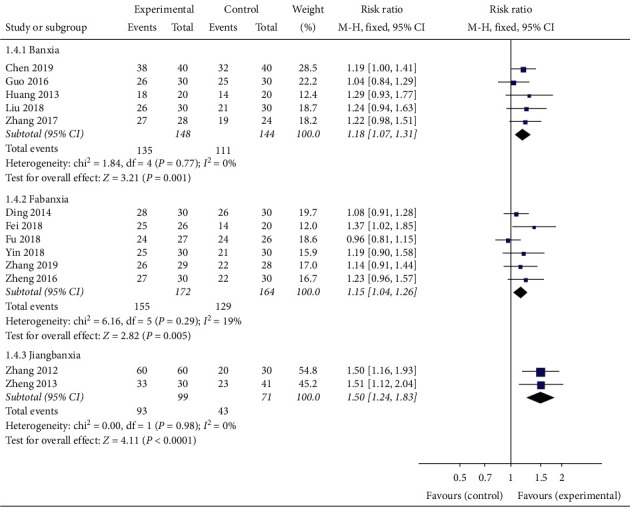
The total effective rate analysis of different proceeding method of Banxia.

In order to evaluate whether the efficacy of Banxia formulae was affected by variable factors, subgroup analyses were conducted according to the course of treatment (≤21 d, >21 d), the dosage of Banxia (≤9 g, >9 g), and the processing method of Banxia (Banxia, Fabanxia, Jiangbanxia). The results of the subgroup analysis showed the total effective rate of the experimental group was significantly higher than that of the control group. The data was listed as follows: treatment duration ≤21 days (*RR* = 1.15, 95% *CI* 1.05 to 1.25, *Z* = 3.18, *P* = 0.001, heterogeneity *χ2* = 6.03, *P* = 0.42, *I*^*2*^ = 1%) (Figure 4), the treatment duration >21 days (*RR* = 1.31, 95% *CI* 1.19 to 1.44, *Z* = 5.64, *P* < 0.00001, heterogeneity *χ2* = 8.71, *P* = 0.19, *I*^*2*^ = 31%) (Figure 4), the dosage of Banxia ≤9 g (*RR* = 1.30, 95% *CI* 1.14 to 1.49, *Z* = 3.88, *P* = 0.0001, heterogeneity *χ2* = 2.62, *P* = 0.45, *I*^*2*^ = 0%) (Figure 5), dosage of Banxia > 9 g (*RR* = 1.21, 95% *CI* 1.12 to 1.30, *Z* = 5.10, *P* < 0.00001, heterogeneity *χ2* = 13.87, *P* = 0.13, *I*^*2*^ = 35%) (Figure 5), Banxia (*RR* = 1.18, 95% *CI* 1.07 to 1.31, *Z* = 3.21, *P* = 0.001, heterogeneity *χ2* = 1.84, *P* = 0.77, *I*^*2*^ = 0%) (Figure 6), Fabanxia (*RR* = 1.15, 95% *CI* 1.04 to 1.26, *Z* = 2.82, *P* = 0.005, *I*^*2*^ = 19%) (Figure 6), and Jiangbanxia (*RR* = 1.50, 95% *CI* 1.24 to 1.83, *Z* = 4.11, *P* < 0.0001, heterogeneity *χ2* = 0.00, *P* = 0.98, *I*^*2*^ = 0%) (Figure 6).

The subgroup analysis did not show any significant differences among the subgroups divided according to treatment duration, the dosage of Banxia, and the processing method of Banxia (Table 4).

#### 3.5.2. Total Score of PSQI

**Figure 7:**
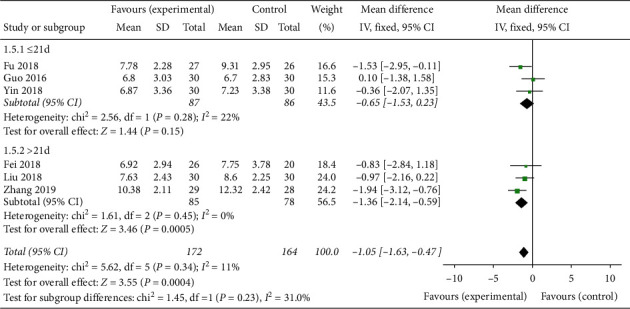
The Pittsburgh Sleep Quality Index (PSQI) total score analysis of different duration.

Six studies reported the total score of PSQI. The results varied with the course of treatment. When the course of treatment ≤21 days, the efficacy on the total score of PSQI of the experimental group was equivalent to that of the control group (*MD* = -0.65, 95% *CI* -1.53 to 0.23, *P* = 0.15 > 0.05, heterogeneity *χ2* = 2.56, *P* = 0.28, *I*^*2*^ = 22%). When the course of treatment > 21 days, the improvement of total score of PSQI in the groups treated by Banxia formulae was better than that in the groups treated by conventional western medicines (*MD* = -1.36, 95% *CI* -2.14 to -0.59, *P* = 0.0005 < 0.05, heterogeneity *χ2* = 1.61, *P* = 0.45, *I*^*2*^ = 0%). According to the integrated data, Banxia formulae had beneficial effects in the treatment of insomnia patients (*MD* = −1.05, 95% CI −1.63 to −0.47, *P* = 0.0004, heterogeneity *χ2* = 5.62, *P* = 0.34, *I*^*2*^ = 11%) (Figure 7).

#### 3.5.3. TCM Syndrome Score

**Figure 8:**
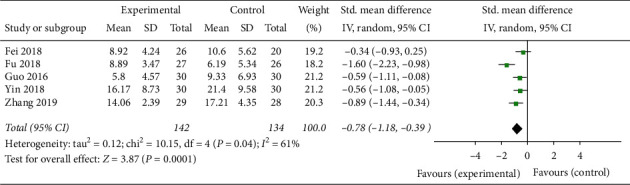
The TCM syndrome score analysis of Banxia formulae for insomnia.

Five studies analyzed the TCM syndrome score. Compared with the control group, the TCM syndrome score of the group treated by Banxia formula was significantly improved (SMD = −0.78, 95% *CI* −1.18 to −0.39, *Z* = 3.87, *P* = 0.0001), and there was a strong heterogeneity (*P* = 0.04, *I*^*2*^ = 61%) which was possibly caused by the difference in the scoring criteria for the TCM syndrome (Figure 8).

### 3.6. Adverse Event(s)

Eight studies mentioned adverse events, in which 5 reported adverse events occurring during treatment and provided sufficient information about the adverse events [20, 23, 26, 29, 32]. A total of 6.5% (10/154) patients in the experimental group and 14.9% (22/148) patients in the control group suffered from adverse events (Table 5). Some patients had multiple events. Three studies declared there were no serious adverse events or side effects in the experimental group and the control group [22, 24, 25].

Adverse events in the experimental groups mainly included numbness in the lips or the tongue, throat discomfort, bitter taste in the mouth, dry mouth, nausea, acid reflux, heartburn, diarrhea, etc. Adverse events in the control group mainly included daytime drowsiness, dizziness, fatigue, dry mouth, bitter taste in the mouth, constipation, upset, drowsiness, deterioration of memory, and loss of appetite. There were no reports of any serious adverse events related to drugs used in the studies.

**Figure 9:**
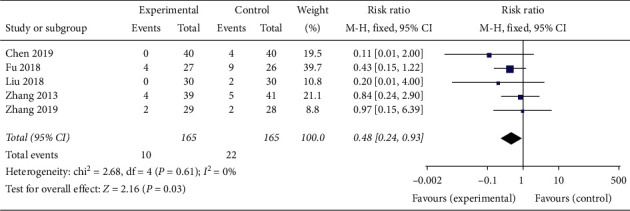
The adverse events analysis of Banxia formulae for insomnia.

Meta-analysis showed that there was statistically significant difference in the incidence of adverse events between the two groups (*RR* = 0.48, 95% *CI* 0.24 to 0.93, *P* = 0.03, heterogeneity *χ2* = 2.68, *P* = 0.61, *I*^*2*^ = 0%) (Figure 9).

### 3.7. Publication Bias

**Figure 10:**
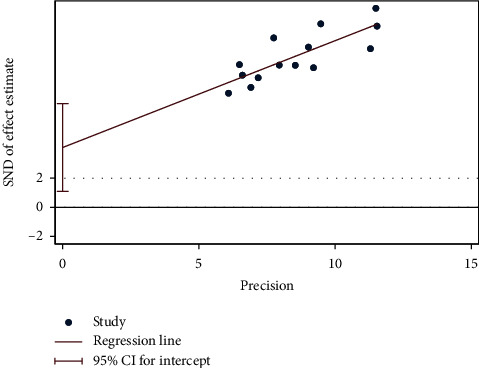
Egger's test of the total effective rate.

In the Egger's test for evaluating publication bias in total effective rate of the two groups, *P* = 0.012, which indicated there was certain publication bias (Figure 10). For studies on the other outcomes, because the number was less than 10, Egger's test was not performed.

### 3.8. Sensitivity Analysis

The sensitivity analysis did not indicate that the final outcome would change because of the exclusion of any individual study, which meant that no single study can significantly affect the pooled *RR* and 95% *CI*.

## 4. Discussion

### 4.1. Summary of Evidence

Banxia is a classic Chinese medicine with a long history of treating insomnia. Many clinical trials and pharmacological studies [34–36] as well as some reviews [37, 38] have provided evidence for the efficacy of Banxia in treatment of insomnia. However, there is no meta-analysis on the value of Banxia formulae for insomnia. This paper is a systematic review of 14 high-quality RCTs including 910 participants aiming at determining the efficacy and safety of Banxia formulae in treatment of insomnia compared with conventional western medicines. Through the study, we found that Banxia formulae provided statistically significant benefits in improving the total effective rate (*RR* = 1.23, 95% *CI* 1.16 to 1.31), the PSQI (*MD* = −1.05, 95% *CI* −1.63 to −0.47) and the TCM syndrome score (*SMD* = −0.78, 95% *CI* −1.18 to −0.39), as well as in decreasing the incidence of adverse events (*RR* = 0.48, 95% *CI* 0.24 to 0.93). The results of this meta-analysis showed Banxia formulae, compared with conventional western medicines, could significantly improve the total effective rate of insomnia patients, regardless of the course of treatment, the dosage, and proceeding method of Banxia. When the course of treatment is longer than 21 days, Banxia formulae can significantly improve the PSQI of insomnia patients.

Although 5 studies including 154 patients reported adverse events, only 10 patients had mild adverse events which was possibly related to Banxia formulae without powerful evidence. The meta-analysis showed that there was statistically significant difference in the incidence of adverse events between the two groups (*RR* = 0.48, 95% *CI* 0.24 to 0.93, *P* = 0.03, heterogeneity *χ2* = 2.68, *P* = 0.61, *I*^*2*^ = 0%). Therefore, current studies indicate the efficacy of Banxia formulae in treatment of insomnia is stable, without adverse reactions.

### 4.2. Limitations

Some limitations existed in this study. Firstly, despite the efforts by us to search the commonly used databases at home and abroad as comprehensively as possible, all the finally included studies were conducted and published in China, because traditional Chinese medicine has not been widely promoted and used in other countries. Therefore, the results of this study have certain limitations. Secondly, although trials with high risk of bias were excluded based on RoB2, some included studies were still short on methodological details. Only two studies described an appropriate method of allocation concealment, and only two studies used single-blind method. Due to the condition limitations, the current designs for RCTs on Banxia formulae could not meet the demand of blind method compared with those for RCTs on conventional therapy. Thirdly, Egger's test (*P* = 0. 012) indicated the existence of publication bias, so the efficacy of Banxia formulae on insomnia may be overstated. Therefore, in this meta-analysis, the efficacy and safety of Banxia formula in the treatment of insomnia are based on the current research data, and more high-quality, multi-center, large sample RCTs are needed for further evaluation.

### 4.3. Implications for Practice

Modern pharmacological studies show that Banxia or Banxia preparations have sedative and hypnotic effects. There are also some studies about the mechanism of Banxia. Wu et al. found that ethanol fraction from Rhizoma Pinelliae Praeparatum possessed sedative, hypnotic, and anticonvulsant activities and these activities may be related to the GABAergic system [36]. Lin et al. reported that Banxia Preparation (raw Banxia processed with liquor rice, lime and alum as adjuvant materials) reduced wakefulness and increased sleep in mice by increasing the number of random eye movement sleep episodes, the number of transitions from non-random eye movement sleep to random eye movement sleep and from random eye movement sleep to wakefulness [34]. Banxia has irritative toxicity to oral cavity, throat, gastrointestinal mucosa, and cardiotoxicity, which can cause vomiting, diarrhea, fetal deformity and death, inflammatory reaction, and liver damage [39–41]. In the *Pharmacopoeia of People's Republic of China*, the prescribed dosage of Banxia is 3-9g [42]. Studies have shown the toxic constituent of Banxia is special acicular crystal which is called raphides. The poisonous raphides are mainly composed of calcium oxalate, proteins, and microamount of polysaccharides [43]. The irritative toxicity of poisonous raphides is related to macrophages [44]. The mechanism is that poisonous raphides penetrate into tissues to activate resident macrophages, release phagocytic and inflammatory cytokines, and cause mass migration of neutrophil, which finally leads to strong acute inflammatory response [45]. Banxia combined with ginger or used in its preparation forms (such as Fabanxia, Jiangbanxia, and Qingbanxia) can reduce the toxicity of Banxia [40, 46, 47].

In the included studies, there were 5 trials with Banxia and 9 trials with Banxia preparation (6 Fabanxia, 2 Jiangbanxia, 1 Qingbanxia). As for the dosage of Banxia, 4 trials were ≤9 g and 10 trials were >9 g, which showed the clinical application habit of Banxia. In this study, we found Banxia formulae can significantly improve the total effective rate of patients with insomnia, regardless of the dosage and processing method of Banxia, and the safety of Banxia formulae for insomnia is relatively stable. However, due to the lack of description of adverse events in the outcome, we did not analyze the safety of Banxia based on its dosage and processing methods, so further study on this aspect is needed.

### 4.4. Implications for Further Study

At first, in order to facilitate more reliable comparison of study results, more clinical trials with large samples which is carefully designed according to international standards are needed. More attention should be paid to the principle of randomization and allocation concealment. Secondly, the standards of outcome measures and test drugs should be as uniform as possible, so as to strengthen the evidence and make the reevaluation more reliable. Finally, in this study, we found Gancao, Chenpi, Fuling, Zhuru, Huanglian, and Dangshen were the most frequently used herbs in Banxia formulae for treating insomnia, which should be considered firstly when formulating optimal Banxia formula for insomnia. At the same time, for the purpose of reducing toxicity, we recommend Banxia should be used with ginger or used in processed forms.

## 5. Conclusion

According to the current studies, the efficacy of Banxia formula in the treatment of insomnia is better than that of the conventional western medicines, and its safety is relatively stable. Current evidence-supported Banxia formulae could be used as a beneficial treatment for insomnia. However, due to the limitations of this study, further research and evaluation are needed.

## Figures and Tables

**Table 1:** Basic characteristics of the included studies.

Included trials treatment	Publication language	No. of participants (male/female; age years)		Treatment		Outcome index	Duration	Intergroup difference
		Experimental	Control	Experimental	Control			
Chen 2019 [20]	Chinese	25/15; 38.8	23/17; 38.6	Huanglian wendan decoction	Tranquillizer	(1) Total effective rate	20 d	(1) *P* < 0.05
						(4) Adverse event(s)		(4)
Ding 2014 [21]	Chinese	12/18; 62.5	13/17; 63.5	Wendan decoction	Tranquillizer	(1) Total effective rate	28 d	(1) *P* < 0.05
Fei 2018 [22]	Chinese	8/18; 40.62	6/14; 41.05	Chaihu wendan decoction	Estazolam	(1) Total effective rate	4 w	(1) *P* < 0.05
						(2) PSQI total score		(2) *P* < 0.05
						(3) TCM syndrome score		(3) *P* < 0.05
						(4) Adverse event(s)		(4)
Fu 2018 [23]	Chinese	10/17; 57.33	8/18; 55.08	Banxia Shumi decoction	Estazolam	(1) Total effective rate	3 w	(1) *P* > 0.05
						(2) PSQI total score		(2) *P* < 0.05
						(3) TCM syndrome score		(3) *P* < 0.05
						(4) Adverse event(s)		(4) *P* > 0.05
Guo 2016 [24]	Chinese	17/13; 42.33	14/16; 40.43	Banxia Shumi decoction	Zopiclone	(1) Total effective rate	21 d	(1) *P* > 0.05
						(2) PSQI total score		(2) *P* < 0.05
						(3) TCM syndrome score		(3) *P* < 0.05
						(4) Adverse event(s)		(4)
Huang 2013 [25]	Chinese	15/5; 36.82	10/10; 41.16	Wendan decoction	Tranquillizer	(1) Total effective rate	20 d	(1) *P* < 0.05
						(4) Adverse event(s)		(4)
Liu 2018 [26]	Chinese	14/16; 52.43	13/17; 51.57	Banxia Xiexin decoction	Alprazolam	(1) Total effective rate	4 w	(1) *P* < 0.05
						(2) PSQI total score		(2) *P* > 0.05
						(4) Adverse event(s)		(4)
Wang 2012 [27]	Chinese	34/28; 68.4	24/26; 69.6	Huoxue Huayu Tongluo decoction	Estazolam	(1) Total effective rate	30 d	(1) *P* < 0.05
Yin 2018 [28]	Chinese	14/16; 24.74	14/16; 24.33	Banxia Xiexin decoction	Estazolam	(1) Total effective rate	21 d	(1) *P* > 0.05
						(2) PSQI total score		(2) *P* > 0.05
						(3) TCM syndrome score		(3) *P* < 0.05
Zhang 2012 [29]	Chinese	21/39; 19–65	10/20; 18–63	Banxia formula	Tranquillizer	(1) Total effective rate	4 w	(1) *P* < 0.05
Zhang 2013 [30]	Chinese	16/23; 23–65	17/24; 22–64	Anshen Huatan decoction	Estazolam	(1) Total effective rate	4 w	(1) *P* < 0.05
Zhang 2017 [31]	Chinese	15/13; 59	12/12; 48	Banxia Shumi decoction and Huanglian wendan decoction	Alprazolam	(1) Total effective rate	2 w	(1) *P* < 0.05
						(4) Adverse event(s)		(4) *P* > 0.05
Zhang 2019 [32]	Chinese	8/21; 49.38	6/22; 44.21	Banxia Xinxie decoction and Huanglian wendan decoction	Lorazepam	(1) Total effective rate	4 w	(1) *P* < 0.05
						(2) PSQI total score		(2) *P* < 0.05
						(3) TCM syndrome score		(3) *P* < 0.05
						(4) Adverse event(s)		(4) *P* > 0.05
Zheng 2016 [33]	Chinese	9/21; 40.13	12/18; 38.25	Banxia Shumi decoction	Tranquillizer	(1) Total effective rate	7 d	(1) *P* < 0.05

PSQI, Pittsburgh Sleep Quality Index.

**Table 2:** The constituent of Banxia formulae in each included study.

Included trials	Formula	Ingredient		Dosage
		Latin name	English name		Chinese name
Chen 2019 [20]	Huanglian wendan decoction	(1) Pinelliae rhizoma	(1) Pinellia ternata	(1) Banxia	15 g
		(2) Caulis bambusae in Taenia	(2) Bamboo shavings	(2) Zhuru	15 g
		(3) Citri reticulatae pericarpium	(3) Tangerine peel	(3) Chenpi	12 g
		(4) Fructus Aurantii immaturus	(4) Immature fruit of trifoliate-orange	(4) Zhishi	10 g
		(5) Coptidis rhizoma	(5) Coptis root, Chinese goldthread	(5) Huanglian	8 g
		(6) Zizyphus jujuba	(6) Chinese date	(6) Dazao	5 g
		(7) Glycyrrhizae radix et rhizoma	(7) Liquorice root	(7) Zhigancao	3 g
		(8) Zingiber officinale rosc.	(8) Ginger	(8) Shengjiang	3 g

Ding 2014 [21]	Wendan decoction	(1) *Cyperus* rotundus *L*.	(1) Nutgrass galingale rhizome	(1) Xiangfu	20 g
		(2) Curcumae radix	(2) Aromatic turmeric root-tuber	(2) Yujin	15 g
		(3) Albiziae cortex	(3) Silktree albizia bark	(3) Hehuanpi	15 g
		(4) Caulis bambusae in Taenia	(4) Bamboo shavings	(4) Zhuru	15 g
		(5) Fructus Aurantii Immaturus	(5) Immature fruit of trifoliate-orange	(5) Zhishi	10 g
		(6) Citri reticulatae pericarpium	(6) Tangerine peel	(6) Chenpi	10 g
		(7) Poria	(7) Indian buead tuckahoe	(7) Fuling	15 g
		(8) Pinelliae rhizoma	(8) Pinellia ternata	(8) Fabanxia	10 g
		(9) Ziziphi Spinosae Semen	(9) Spine date seed	(9) Suanzaoren	15 g
		(10) Polygalae radix	(10) Thinleaf milkwort root	(10) Zhiyuanzhi	10 g
		(11) Caulis polygoni multiflori	(11) Tuber fleeceflower stem	(11) Yejiaoteng	15 g

Fei 2018 [22]	Chaihu wendan decoction	(1) Radix bupleuri	(1) Chinese thorowax root	(1) Chaihu	10 g
		(2) Pinelliae rhizoma	(2) Pinellia ternata	(2) Fabanxia	10 g
		(3) Scutellariae radix	(3) Baical skullcap root	(3) Huangqin	10 g
		(4) Codonopsis radix	(4) Tangshen	(4) Dangshen	10 g
		(5) Aurantii fructus	(5) Immature trifoliate-orange fruit	(5) Zhiqiao	10 g
		(6) Citri reticulatae pericarpium	(6) Tangerine peel	(6) Chenpi	10 g
		(7) Caulis bambusae in Taenia	(7) Bamboo shavings	(7) Zhuru	10 g
		(8) Poria	(8) Indian buead tuckahoe	(8) Fuling	10 g
		(9) Zingiber officinale rosc.	(9) Ginger	(9) Shengjiang	5 g
		(10) Zizyphus jujuba	(10) Chinese date	(10) Hongzao	10 g
		(11) Glycyrrhizae radix et rhizoma	(11) Liquorice root	(11) Zhigancao	5 g

Fu 2018 [23]	Banxia Shumi decoction	(1) Pinelliae rhizoma	(1) Pinellia ternata	(1) Fabanxia	30 g
		(2) Setarie italica	(2) Husked sorghum	(2) Shumi	15 g
		(3) Poria	(3) Indian buead tuckahoe	(3) Fuling	20 g
		(4) Codonopsis radix	(4) Tangshen	(4) Dangshen	15 g
		(5) Ganoderma	(5) Lucid ganoderma	(5) Lingzhi	15 g
		(6) Albiziae cortex	(6) Silktree albizia bark	(6) Hehuanpi	15 g
		(7) Polygalae radix	(7) Thinleaf milkwort root	(7) Yuanzhi	15 g
		(8) Acori Tatarinowii rhizoma	(8) Acorus tatarinowii	(8) Shichangpu	10 g
		(9) Aurantii fructus	(9) Immature trifoliate-orange fruit	(9) Zhiqiao	15 g
		(10) Glycyrrhizae radix et rhizoma	(10) Liquorice root	(10) Gancao	5 g

Guo 2016 [24]	Banxia Shumi decoction	(1) Pinelliae rhizoma	(1) Pinellia ternata	(1) Banxia	15 g
		(2) Coicis Semen	(2) Ma-yuen jobstears seed	(2) Yiyiren	30 g
		(3) Coptidis rhizoma	(3) Coptis root, Chinese goldthread	(3) Huanglian	5 g
		(4) Prunellae Spica	(4) Common selfheal spike	(4) Xiakucao	15 g
		(5) Plumula nelumbinis	(5) Lotus plumule	(5) Lianzixin	5 g
		(6) Ziziphi Spinosae Semen	(6) Spine date seed	(6) Suanzaoren	30 g
		(7) Albiziae cortex	(7) Silktree albizia bark	(7) Hehuanpi	10 g
		(8) Caulis polygoni multiflori	(8) Tuber fleeceflower stem	(8) Shouwuteng	30 g

Huang 2013 [25]	Wendan decoction	(1) Citri reticulatae pericarpium	(1) Tangerine peel	(1) Chenpi	9 g
		(2) Pinelliae rhizoma	(2) Pinellia ternata	(2) Banxia	6 g
		(3) Polygalae radix	(3) Thinleaf milkwort root	(3) Yuanzhi	3 g
		(4) Caulis bambusae in Taenia	(4) Bamboo shavings	(4) Zhuru	6 g
		(5) Flos albiziae	(5) Silktree albizzia flower	(5) Hehuanhua	9 g
		(6) Poria	(6) Indian buead tuckahoe	(6) Fuling	5 g
		(7) Curcumae radix	(7) Aromatic turmeric root-tuber	(7) Yujin	6 g
		(8) Glycyrrhizae radix et rhizoma	(8) Liquorice root	(8) Zhigancao	3 g
		(9) Schisandrae chinensis fructus	(9) Chinese magnolcavine fruit	(9) Wuweizi	6 g
		(10) Acorus calamus	(10) *Calamus*	(10) Changpu	6 g

Liu 2018 [26]	Banxia Xiexin decoction	(1) Pinelliae rhizoma	(1) Pinellia ternata	(1) Banxia	6 g
		(2) Scutellariae radix	(2) Baical skullcap root	(2) Huangqin	9 g
		(3) Coptidis rhizoma	(3) Coptis root, Chinese goldthread	(3) Huanglian	3 g
		(4) Codonopsis radix	(4) Tangshen	(4) Dangshen	15 g
		(5) Zingiberis rhizoma	(5) Dried ginger	(5) Ganjiang	6 g
		(6) Glycyrrhizae radix et rhizoma	(6) Liquorice root	(6) Gancao	6 g
		(7) Magnoliae officinalis cortex	(7) Officinal magnolia bark	(7) Houpo	9 g
		(8) Prunellae Spica	(8) Common Selfheal spike	(8) Xiakucao	12 g
		(9) Coicis Semen	(9) Ma-yuen jobstears seed	(9) Yiyiren	24 g
		(10) Zizyphus jujuba	(10) Chinese date	(10) Dazao	3

Wang 2012 [27]	Banxia formula	(1) Pinelliae rhizoma	(1) Pinellia ternata	(1) Qingbanxia	12 g
		(2) Citri reticulatae pericarpium	(2) Tangerine peel	(2) Chenpi	10 g
		(3) Poria	(3) Indian buead tuckahoe	(3) Fuling	20 g
		(4) Arisaema cum bile	(4) Bile arisaema	(4) Dannanxing	8 g
		(5) Polygalae radix	(5) Thinleaf milkwort root	(5) Yuanzhi	12 g
		(6) Acori Tatarinowii rhizoma	(6) Acorus tatarinowii	(6) Shichangpu	12 g
		(7) Radix salviae miltiorrhizae	(7) Danshen root	(7) Danshen	30 g
		(8) Rhizoma ligustici chuanxiong	(8) Sichuan lovage rhizome	(8) Chuanxiong	9 g
		(9) Paeoniae radix rubra	(9) Red paeony root	(9) Chishao	15 g
		(10) Ziziphi Spinosae Semen	(10) Spine date seed	(10) Suanzaoren	30–60 g
		(11) Spatholobi caulis	(11) Suberect Spatholobus stem	(11) Jixueteng	30 g
		(12) Caulis polygoni multiflori	(12) Tuber fleeceflower stem	(12) Shouwuteng	30 g
		(13) Glycyrrhizae radix et rhizoma	(13) Liquorice root	(13) Gancao	10 g

Yin 2018 [28]	Banxia Xiexin decoction	(1) Mori cortex	(1) White mulberry root-bark	(1) Sangbaipi	10 g
		(2) Pinelliae rhizoma	(2) Pinellia ternata	(2) Fabanxia	10 g
		(3) Zingiberis rhizoma	(3) Dried ginger	(3) Ganjiang	3–10 g
		(4) Coptidis rhizoma	(4) Coptis root, Chinese goldthread	(4) Huanglian	3-5 g
		(5) Scutellariae radix	(5) Baikal skullcap root	(5) Huangqin	10 g
		(6) Pogostemonis herba	(6) Cablin potchouli herb	(6) Huoxiang	10 g
		(7) Atractylodis rhizoma	(7) Rhizoma atractylodis	(7) Cangzhu	10 g
		(8) Magnoliae officinalis cortex	(8) Officinal magnolia bark	(8) Houpo	10 g
		(9) Poria	(9) Indian buead tuckahoe	(9) Fuling	10 g
		(10) Pulvis Talci	(10) Talc powder	(10) Huashi	20 g
		(11) Medulla Tetrapanacis	(11) Ricepaperplant pith	(11) Tongcao	10 g
		(12) Codonopsis radix	(12) Tangshen	(12) Dangshen	10 g
		(13) Glycyrrhizae radix et rhizoma	(13) Liquorice root	(13) Gancao	3 g

Zhang 2012 [29]	Huanglian wendan decoction	(1) Coptidis rhizoma	(1) Coptis root, Chinese goldthread	(1) Huanglian	9 g
		(2) Caulis bambusae in Taenia	(2) Bamboo shavings	(2) Jiangzhuru	9 g
		(3) Arisaema cum bile	(3) Bile arisaema	(3) Dannanxing	9 g
		(4) Pinelliae rhizoma	(4) Pinellia ternata	(4) Jiangbanxia	9 g
		(5) Citri reticulatae pericarpium	(5) Tangerine peel	(5) Chenpi	9 g
		(6) Fructus Aurantii Immaturus	(6) Immature fruit of trifoliate-orange	(6) Zhishi	12 g
		(7) Poria	(7) Indian buead tuckahoe	(7) Fushen	15 g
		(8) Gardenia jasminoides ellis	(8) Cape jasmine	(8) Jiaozhizi	12 g
		(9) Hyriopsis cumingii(Lea) or cristaria plicata(Leach) or pteria martensii(Dunker)	(9) Mother-of-pearl	(9) Zhenzhumu	30 g
		(10) Glycyrrhizae radix et rhizoma	(10) Liquorice root	(10) Zhigancao	6 g
		(11) Zingiber officinale rosc.	(11) Ginger	(11) Shengjiang	6 g
		(12) Zizyphus jujuba	(12) Chinese date	(12) Dazao	10 g

Zhang 2013 [30]	Anshen Huatan decoction (free-frying Chinese medicine granule)	(1) Pinelliae rhizoma	(1) Pinellia ternata	(1) Jiangbanxia	10 g
		(2) Caulis polygoni multiflori	(2) Tuber fleeceflower stem	(2) Shouwuteng	30 g
		(3) Citri reticulatae pericarpium	(3) Tangerine peel	(3) Chenpi	12 g
		(4) Poria	(4) Indian buead tuckahoe	(4) Fushen	20 g
		(5) Aurantii fructus	(5) Immature trifoliate-orange fruit	(5) Zhiqiao	12 g
		(6) Caulis bambusae in Taenia	(6) Bamboo shavings	(6) Zhuru	10 g
		(7) Albiziae cortex	(7) Silktree albizia bark	(7) Hehuanpi	30 g
		(8) Os draconis	(8) Dragon's bones	(8) Longgu	20 g
		(9) Ostreae concha	(9) Common oyster shell	(9) Muli	20 g
		(10) Glycyrrhizae radix et rhizoma	(10) Liquorice root	(10) Gancao	6 g
		(11) Ziziphi Spinosae Semen	(11) Spine date seed	(11) Suanzaoren	30 g
		(12) Cortex magnoliae officinalis	(12) Officinal magnolia bark	(12) Hupo	9 g

Zhang 2017 [31]	Banxia Shumi decoction and Huanglian wendan decoction	(1) Pinelliae rhizoma	(1) Pinellia ternata	(1) Banxia	15 g
		(2) Setarie italica	(2) Husked sorghum	(2) Shumi	15 g
		(3) Citri reticulatae pericarpium	(3) Tangerine peel	(3) Chenpi	9 g
		(4) Poria	(4) Indian buead tuckahoe	(4) Fuling	12 g
		(5) Fructus AIIurantii Immaturus	(5) Immature fruit of trifoliate-orange	(5) Zhishi	10 g
		(6) Coptidis rhizoma	(6) Coptis root, Chinese goldthread	(6) Huanglian	5 g
		(7) Caulis Bambusae in Taenia	(7) Bamboo shavings	(7) Zhuru	12 g
		(8) Massa medicata fermentata	(8) Medicated leaven	(8) Shenqu	15 g

Zhang 2019 [32]	Banxia Xiexin decoction and Huanglian wendan decoction	(1) Pinelliae rhizoma	(1) Pinellia ternata	(1) Fabanxia	9 g
		(2) Os draconis	(2) Dragon's bones	(2) Longgu	30 g
		(3) Ostreae concha	(3) Common oyster shell	(3) Muli	30 g
		(4) Coptidis rhizoma	(4) Coptis root, Chinese goldthread	(4) Huanglian	6 g
		(5) Scutellariae radix	(5) Baical skullcap root	(5) Huangqin	12 g
		(6) Acori Tatarinowii rhizoma	(6) Acorus tatarinowii	(6) Shichangpu	10 g
		(7) Polygalae radix	(7) Thinleaf milkwort root	(7) Yuanzhi	10 g
		(8) Citri reticulatae pericarpium	(8) Tangerine peel	(8) Chenpi	10 g
		(9) Fructus AIIurantii Immaturus	(9) Immature fruit of trifoliate-orange	(9) Zhishi	10 g
		(10) Caulis bambusae in Taenia	(10) Bamboo shavings	(10) Zhuru	10 g
		(11) Poria	(11) Indian buead tuckahoe	(11) Fuling	10 g
		(12) Paeoniae radix alba	(12) White paeony root	(12) Baishao	15 g
		(13) Codonopsis radix	(13) Tangshen	(13) Dangshen	10 g
		(14) Glycyrrhizae radix et rhizoma	(14) Liquorice root	(14) Gancao	6 g
		(15) Zizyphus jujuba	(15) Chinese date	(15) Dazao	3

Zheng 2016 [33]	Banxia Shumi decoction	(1) Pinelliae rhizoma	(1) Pinellia ternata	(1) Fabanxia	40 g
		(2) Poria	(2) Indian buead tuckahoe	(2) Fuling	30 g
		(3) *Dioscorea* opposita	(3) Common yam rhizome	(3) Shanyao	30 g
		(4) Albiziae cortex	(4) Silktree albizia bark	(4) Hehuanpi	30 g
		(5) Os draconis	(5) Dragon's bones	(5) Longgu	18 g
		(6) Ostreae concha	(6) Common oyster shell	(6) Muli	18 g
		(7) Caulis polygoni multiflori	(7) Tuber fleeceflower stem	(7) Shouwuteng	30 g
		(8) Codonopsis radix	(8) Tangshen	(8) Dangshen	12 g
		(9) Atractylodis macrocephalae rhizoma	(9) Largehead atractylodes Rh	(9) Baizhu	10 g

**Table 3:** The top 7 frequency Chinese herbal medicines of formulae.

English name	Chinese name	Frequency	The total frequency (%)
Pinellia ternata	Banxia	14	100.0
Liquorice root	Gancao	10	71.4
Tangerine peel	Chenpi	9	64.3
Indian buead tuckahoe	Fuling	9	64.3
Bamboo shavings	Zhuru	8	57.1
Coptis root, Chinese goldthread	Huanglian	7	50.0
Tangshen	Dangshen	6	42.9

**Table 4:** Subgroup analysis of the total effective rate for each variable.

Variable	Effects model	RR	95% CI	Figure	*P* value	Heterogeneity (chi-squared test and *I*^2^ statistic)	Total patients
*Duration*							
≤21 d	Fixed	1.15	1.05–1.25	4	0.001	*P* = 0.42, *I*^2^ = 1%	405
＞21 d	Fixed	1.31	1.19–1.44	4	＜0.00001	*P* = 0.19, *I*^2^ = 31%	505

*The dose of Banxia*							
≤9 g	Fixed	1.30	1.14–1.49	5	0.0001	*P* = 0.45, *I*^2^ = 0%	247
＞9 g	Fixed	1.21	1.12–1.30	5	＜0.00001	*P* = 0.13, *I*^2^ = 35%	663

*The proceeding method of Banxia*							
Banxia	Fixed	1.18	1.07–1.31	6	0.001	*P* = 0.77, *I*^2^ = 0%	292
Fabanxia	Fixed	1.15	1.04–1.26	6	0.005	*P* = 0.29, *I*^2^ = 19%	336
Jiangbanxia	Fixed	1.50	1.24–1.83	6	＜0.0001	*P* = 0.98, *I*^2^ = 0%	170

**Table 5:** Summary of adverse events.

Studies	Experimental		Control		Adverse events	
	*n* (Case)	Total	*n* (Case)	Total	Experimental	Control
Chen 2019 [20]	0	40	4	40		Fatigue, drowsiness, and loss of memory
Fu 2018 [23]	4	27	9	26	Lip numbness, consciously thickened lips, tongue numbness and discomfort in throat, and bitter mouth	Drowsiness during the day, dizziness and fatigue, dry mouth, and bitter mouth
Liu 2018 [26]	0	30	2	30		Constipation
Zhang 2017 [29]	4	28	5	24	Dry mouth, disgusting	Dizziness, fatigue, upset, and loss of appetite
Zhang 2019 [32]	2	29	2	28	Acid reflux, heartburn, and loose stools	Dizzy
